# Including transgender populations in mathematical models for HIV treatment and prevention: current barriers and policy implications

**DOI:** 10.1002/jia2.26304

**Published:** 2024-06-12

**Authors:** Diana M. Tordoff, Arjee Restar, Brian Minalga, Atlas Fernandez, Dobromir Dimitrov, Ann Duerr, the Seattle Trans and Nonbinary Sexual Health (STARS) Advisory Board

**Affiliations:** ^1^ Department of Epidemiology University of Washington Seattle Washington USA; ^2^ School of Medicine Stanford University Palo Alto California USA; ^3^ Fred Hutchinson Cancer Center Seattle Washington USA; ^4^ Building Changes Seattle Washington USA; ^5^ Department of Applied Mathematics University of Washington Seattle Washington USA; ^6^ Department of Global Health University of Washington Seattle Washington USA

**Keywords:** HIV, mathematical modelling, epidemic modelling, transgender, nonbinary, HIV policy

## Abstract

**Introduction:**

Mathematical models of HIV have been uniquely important in directing and evaluating HIV policy. Transgender and nonbinary people are disproportionately impacted by HIV; however, few mathematical models of HIV transmission have been published that are inclusive of transgender and nonbinary populations. This commentary discusses current structural challenges to developing robust and accurate trans‐inclusive models and identifies opportunities for future research and policy, with a focus on examples from the United States.

**Discussion:**

As of April 2024, only seven published mathematical models of HIV transmission include transgender people. Existing models have several notable limitations and biases that limit their utility for informing public health intervention. Notably, no models include transgender men or nonbinary individuals, despite these populations being disproportionately impacted by HIV relative to cisgender populations. In addition, existing mathematical models of HIV transmission do not accurately represent the sexual network of transgender people. Data availability and quality remain a significant barrier to the development of accurate trans‐inclusive mathematical models of HIV. Using a community‐engaged approach, we developed a modelling framework that addresses the limitations of existing model and to highlight how data availability and quality limit the utility of mathematical models for transgender populations.

**Conclusions:**

Modelling is an important tool for HIV prevention planning and a key step towards informing public health interventions, programming and policies for transgender populations. Our modelling framework underscores the importance of accurate trans‐inclusive data collection methodologies, since the relevance of these analyses for informing public health decision‐making is strongly dependent on the validity of the model parameterization and calibration targets. Adopting gender‐inclusive and gender‐specific approaches starting from the development and data collection stages of research can provide insights into how interventions, programming and policies can distinguish unique health needs across all gender groups. Moreover, in light of the data structure limitations, designing longitudinal surveillance data systems and probability samples will be critical to fill key research gaps, highlight progress and provide additional rigour to the current evidence. Investments and initiatives like Ending the HIV Epidemic in the United States can be further expanded and are highly needed to prioritize and value transgender populations across funding structures, goals and outcome measures.

## 1 INTRODUCTION

Transgender and nonbinary people, especially Black, Latina and Indigenous transgender women in the Americas, are disproportionately impacted by HIV [1, 2]. An estimated 0.6% of the population is transgender, or at least 1.6 million adults and adolescents in the United States [3, 4] and 32 million worldwide. Globally, it is estimated that transgender women have 66 times the odds and transgender men have 6.8 times the odds of living with HIV compared to the overall adult population [5]. Yet, transgender people experience significant barriers to engaging in healthcare, including HIV prevention and treatment, due to structural barriers rooted in cissexism, anti‐transgender stigma, violence, criminalization, legal recognition and fear of mistreatment in healthcare settings [6, 7, 8, 9]. Consequently, transgender people report low levels of pre‐exposure prophylaxis (PrEP) use and viral suppression relative to cisgender populations [10, 11, 12, 13].

Efforts to end the HIV pandemic will fail without including and accounting for the needs and lived experiences of transgender people. Trans‐inclusive and trans‐specific prevention and treatment interventions are and will continue to be critical components of HIV care; our HIV policy and research tools must be inclusive of transgender people. Mathematical modelling has been a particularly useful and influential tool for understanding epidemic dynamics and planning public health interventions for HIV [14]. Mathematical models of HIV have been uniquely important in directing and evaluating HIV policy, including the development of the UNAIDS 90‐90‐90 targets, identifying combination interventions needed to achieve the US Ending the HIV Epidemic (EHE)’s incidence reduction goals and informing WHO recommendations for PrEP use [15, 16, 17, 18, 19, 20]. Although transgender people are significantly impacted by HIV, few mathematical models of HIV transmission have been published that are inclusive of transgender populations [21, 22, 23, 24, 25, 26, 27].

Although trans‐inclusive mathematical models are essential for HIV treatment and prevention, there are structural challenges to developing these models. This commentary discusses current barriers to developing robust and accurate trans‐inclusive models. Our examples largely focus on the US context. To date, a disproportionate amount of research on transgender populations has been conducted in the United States and the global north [5, 28], therefore, the barriers and challenges discussed herein are likely significantly exacerbated in other global settings. In addition, we undertook a brief modelling exercise to highlight current limitations and identify opportunities for future research and policy.

## 2 DISCUSSION

### 2.1 Utility of mathematical models for transgender health equity

Multilevel interventions are needed to address the structural barriers to accessing HIV prevention and treatment for transgender people [29, 30, 31]. For example, despite high reported willingness to take PrEP among transgender women and men, PrEP uptake has been low among transgender populations [32, 33, 34, 35]. Qualitative studies have identified trans‐specific barriers to PrEP, including concerns that PrEP may interact with hormone therapy, the absence of trans‐inclusive marketing and public health campaigns, stigma, and medical distrust [36, 37, 38, 39, 40, 41]. Transgender participants also cite barriers that are not trans‐specific, such as low awareness of PrEP, difficulty taking daily pills, side effects and cost [42]. Other studies have demonstrated that transgender people recently diagnosed with HIV are also significantly less likely to be linked to care and achieve viral suppression [10, 43].

An unmet need for gender‐affirming healthcare also hinders access to HIV treatment and prevention tools for transgender people [44]. Gender‐affirming healthcare—including access to gender‐affirming hormones, surgeries, as well as inclusive and affirming mental health services—is a critical determinant of health and preventative care [45]. Therefore, delivery of HIV prevention and treatment alongside gender‐affirming healthcare, including access to gender‐affirming hormones, is likely to be effective at increasing HIV testing, antiretroviral therapy (ART) and PrEP uptake and adherence [46, 47, 48]. Several studies have demonstrated that access to gender‐affirming care is associated with higher rates of linkage, retention and viral suppression among transgender women living with HIV [49, 50, 51]. Further, qualitative studies based in the United States have also demonstrated that fear of mistreatment in medical settings and competing priorities for accessing hormones are both barriers to PrEP uptake among transgender women. Co‐administration of hormone therapy alongside PrEP has been shown to significantly improve knowledge, acceptability, uptake and retention for PrEP [52]. Thus, receiving HIV treatment and prevention from trans‐competent providers with training in healthcare for transgender people may be critical for engagement in care, maintaining viral suppression, and promoting PrEP uptake and adherence [33, 36].

### 2.2 Limitations of existing mathematical models

As of writing this article in April 2024, a review of the published literature identified only seven mathematical models of HIV transmission that include transgender people (Table 1A). Existing models have several notable limitations and biases that limit their utility for informing public health interventions. All models include transgender women, and no models include transgender men or nonbinary individuals, despite these populations being disproportionately impacted by HIV relative to cisgender populations. Most models (*n* = 5) are calibrated to local epidemics in Lima, Peru and/or San Francisco, California [21, 22, 23, 24, 25, 26, 27]. Notably, two of these models inappropriately aggregate transgender women with cisgender men who have sex with men, while other models specifically focus on transgender women sex workers and their clients.

**Table 1:** Existing models and data related to HIV among transgender populations

A. Overview of mathematical models of HIV that include transgender women		
Author/citation	Geographic location	Model description
Gomez et al. [22]	Lima, Peru	Men who mostly have sex with women, men who mostly have sex with men, cisgender women sex workers and transgender women.
Poteat et al. [21]	Lima, Peru and San Francisco, CA	Transgender women sex workers not in a stable relationship, transgender women sex workers in a stable relationship, cisgender male clients, cisgender male stable partners.
Melesse et al. [23]	Pakistan	Cisgender women sex workers, people who inject drugs (PWID) and *hijra*/transgender/male sex workers aggregated as one group.
Dimitrov et al. [24]	Lima, Peru	Men who have sex with men (MSM) and transgender women aggregated as one population. Model is calibrated to data on cisgender MSM only.
Bórquez et al. [25]	Lima, Peru	Transgender women sex workers not in a stable relationship, transgender women sex workers in a stable relationship, cisgender male clients, cisgender male stable partners. Model adapted from Poteat et al. (2015).
Bórquez et al. [26]	Lima, Peru	Gay/homosexual self‐identified MSM, heterosexual/bisexual self‐identified MSM, male sex workers and transgender women. Model adapted from Gomez et al. (2012)
Brown et al. [27]	Thirteen Asian countries included in the AIDS Epidemic Model (AEM)	Key populations (MSM, cisgender men and cisgender women sex workers, PWID), including transgender women who: sell sex, have casual partners and have regular partners.

B. Key data sources on HIV prevalence among transgender and nonbinary people in the United States					
Data sources	Year(s) of data collection	Measure	Transgender women	Transgender men	Nonbinary people
Becasen et al Meta‐analysis [1]	2001−2015	HIV Prevalence, % (95% CI)	18.8% (14.9−23.5%)	2.0% (1.0−4.0%)	N/A
US Transgender Population Health (TransPop) Survey [56]	2016−2018	HIV Prevalence, % (95% CI)	6.5% (2.5−16.1%)	0.8% (0.1−5.6%)	5.1% (1.3−18.4%)
US Transgender Survey [6]	2015	HIV Prevalence, %	3.4%	0.3%	0.4%
CDC National HIV Surveillance Report [58]	2014−2020	HIV Prevalence, *n*	10,507−11,949	419−509	182−243

Existing mathematical models of HIV transmission among transgender women also do not accurately represent the sexual network of transgender people. They presume that transgender women exclusively partner with cisgender men, despite evidence that transgender people are diverse in their sexual orientation and choice of sexual and romantic partners. Recent data from the United States show that although most heterosexual transgender women partner with cisgender men, the majority of transgender women do not identify as heterosexual and only 40% of sexual minority transgender women (i.e. who identify as lesbian, gay, bisexual, pansexual and/or queer) reported sex with a cisgender man in the past year [53]. Transgender women also frequently report partnering with cisgender women and as many as one‐third reported having trans‐trans partnerships within the last year. Mathematical models that assume transgender women only partner with cisgender men thus, are only able to inform public health interventions for a small subset of the overall transgender population. The significance of sexual mixing on HIV transmission is amplified by differences in HIV prevalence and HIV prevention utilization among different populations who partner with transgender people [53].

### 2.3 Barriers to developing trans‐inclusive mathematical models

The development of accurate mathematical models of HIV strongly depends on data availability and quality–both for model parameterization and calibration. The inclusion of transgender people in mathematical models requires accurate and longitudinally available data in order to be useful and informative for HIV policies.

The majority of data on transgender populations are from convenience or clinical samples, which may have biased estimates of HIV prevalence [1]. A meta‐analysis of studies conducted in the United States estimated an HIV prevalence of 18.8% among transgender women and 2.0% among transgender men (with no mention of nonbinary people) [1]. The authors note that their meta‐analysis may overestimate HIV prevalence because nearly half of the included studies “recruited from locations or for reasons that would indicate higher‐than‐normal risk for HIV.”[1] In contrast, data from the 2015 US Transgender Survey estimated that HIV prevalence was 3.4% among trans women, 0.3% among trans men and 0.4% among nonbinary people (Table 1B) [6]. While the US Transgender Survey is the largest study of transgender health conducted to date (with over 27,000 participants in 2015 and over 92,000 participants in 2022), it is an online convenience sample and, therefore, may also be subject to sample bias, particularly overrepresentation of White participants. Both programmatic and community‐based survey data are thus vulnerable to different sources of bias, which need to be carefully considered in mathematical models. In contrast, probability samples and surveillance data have the strongest potential for generating population‐based data that are useful for mathematical models.

To date, only two national probability samples in the United States have collected data on transgender identities: the Behavioral Risk Factor Surveillance System (BRFSS) and the US Transgender Population Health (TransPop) Survey [54, 55]. Of these, only the TransPop survey has assessed self‐reported HIV positivity among transgender individuals. This study found that self‐reported HIV positivity was 6.5% among transgender women, 0.8% among transgender men and 5.1% among nonbinary people [56]. These estimates are much higher than what is found in the overall population, underscoring that transgender people are disproportionately impacted by HIV. However, these estimates are much lower than those estimated from meta‐analyses based on convenience samples and clinical data.

Historically, HIV surveillance data on transgender populations have been limited by the inconsistent collection and reporting of gender identity data across local jurisdictions. For example, although 83% of local public health jurisdictions in the United States collected data on transgender identities through their confidential reporting forms, only 15% reported data separately for transgender men and transgender women in their HIV Surveillance Reports [57]. The Centers for Disease Control and Prevention (CDC) first reported disaggregated HIV surveillance data for transgender people in their 2018 HIV surveillance report [58]. Thus, surveillance data on prevalence and incident HIV diagnoses for transgender people in the United States are only available dating back to 2014, and data on engagement in care (e.g. viral suppression) are only available starting in 2018. Although other large national data sources in the United States, such as the federally funded Ryan White HIV/AIDS Program for low‐income individuals living with HIV, provide data on engagement in care and viral suppression for transgender clients overall, these data are not disaggregated for transgender men, transgender women and nonbinary populations [59, 60].

HIV surveillance data are likely significantly undercounting HIV diagnoses among transgender people due to misclassification. According to the CDC's 2020 HIV Surveillance report, 11,949 transgender women, 509 transgender men and 243 nonbinary people in the United States were living with HIV in 2020. This misclassification may be most significant for nonbinary people, since this count appears to be inconsistent (by several orders of magnitude) with prevalence estimates from the TransPop study (i.e. 5.1%) applied to recent conservative estimates of the size of the adult nonbinary population in the United States (i.e. 341,800) [3, 56].

### 2.4 Insights from a modelling framework

We developed a modelling framework to highlight how data availability and quality limit the utility of trans‐inclusive mathematical models. We developed a novel mathematical model of HIV transmission in the United States that more accurately represents transgender people and their sexual network. This model was developed using a community‐engaged approach, where members of the Seattle Trans and Nonbinary Sexual Health (STARS) Advisory Board informed key model assumptions and structure. This model aimed to address several limitations of existing models through its inclusion of transgender men and nonbinary people and by factoring in diverse sexual partnerships among all demographic groups (including trans‐trans partnerships).

We chose to calibrate our model to the best available longitudinal data in the United States: 2015−2020 National HIV Surveillance data on HIV prevalence, ART use and viral suppression reported by the CDC. However, our analysis demonstrated that due to the limitations of these data, model projections differ significantly from empirical data and thus have limited utility for public health intervention planning. In this modelling exercise, we simulated the potential impact of increasing utilization of HIV prevention tools (specifically, access to PrEP and more frequent HIV testing) among transgender people in the United States, in order to achieve the US EHE goals of a 90% reduction in HIV incidence by 2030 [61]. Detailed information about the mathematical model parameterization and calibration are available at https://github.com/dianatordoff/transinclusivemathmodels.

Based on CDC data, we estimated that national HIV incidence in 2020 was 0.36 per 1000 person‐years among transgender women, 0.22 per 1000 person‐years among transgender men and 0.42 per 1000 person‐years among nonbinary people. Notably, the incidence estimated from our model is several orders of magnitude lower than empirical data from the LITE Study, a recent longitudinal cohort of 1312 transgender women living in the six cities in the south and northeastern United States, which found an HIV incidence of 5.5 (95% CI 2.7−8.3) per 1000 person‐years among transgender women between 2018 and 2020 [62]. Based on these incidence rates estimated from our model, we projected that 3096 transgender and nonbinary people in the United States will acquire HIV by 2030, an estimate that is very likely an underestimate. Due to the artificially low incidence rate assumed based on our calibration targets, our model also incorrectly estimated that both HIV testing and PrEP provided only marginal increases in the number and percentage of HIV acquisitions that were prevented over the 10‐year intervention period. For example, in an optimistic scenario, a 100% increase in the rate of both HIV testing and PrEP uptake among all transgender and nonbinary people was estimated to prevent only 25%, 10% and 17% of the new HIV acquisitions between 2020 and 2030 among transgender women, transgender men and nonbinary people, respectively.

Thus, the calibration and outputs of our model were severely limited by the quality of data used for calibration targets. Most notably, due to the limited availability of longitudinal data on HIV prevalence for transgender people, our model assumed that the HIV prevalence reported by the CDC's National HIV Surveillance report was accurate. In actuality, these numbers are likely a gross underestimate of the true number of transgender people living with HIV, especially for transgender men and nonbinary people, who are likely misclassified as cisgender in US surveillance data. Therefore, the absolute reductions and HIV incidence rates estimated in our study are also likely significant underestimates of the impact of the intervention scenarios considered.

## 3 CONCLUSIONS

Modelling is an important tool for HIV prevention planning and a key step towards informing public health interventions, programming and policies for transgender populations. Our modelling exercise underscores the importance of accurate gender‐inclusive data collection methodologies and reporting in HIV/STI surveillance that recognize transgender identities in data systems [63], since the relevance of these analyses for informing public health decision‐making is strongly dependent on the validity of data used for model parameterization and calibration targets.

There are several policy and research implications of using advanced modelling techniques in transgender health research. Adopting gender‐inclusive and gender‐specific approaches starting from the development and data collection stages of research can provide insights into how interventions, programming and policies can distinguish shared and unique health needs, across all genders and specifically within gender groups [44, 45]. Given the recent and unprecedented legislation seeking to criminalize and erode the rights of transgender people in the United States and beyond [64], collecting these data at the local, state and federal levels also necessitates implementing best practices that are in the interest of transgender populations, including being transparent about policies that provide maximum protection of confidentiality and privacy. This will involve the alignment of policies across all levels, including at the facility level, that govern both public and private data security systems, data sharing regulations and data disclosures, to ensure that transgender populations’ personal and health information remain fully protected. Involving stakeholders across areas of policy/legal communities, medical/public health leadership, data security operations, along with transgender communities and trans‐led organizations will be key to ensuring that the development and implementation of surveillance systems are grounded for, by and with transgender communities [65, 66].

Moreover, in light of the data structure limitations identified by our modelling framework, designing longitudinal surveillance data systems and probability samples will be critical to fill key research gaps, highlight progress and provide additional rigour to the current evidence. This includes ensuring that such data systems are able to capture, include or be linked to other systems that measure unique multilevel challenges and barriers faced by transgender populations when accessing HIV prevention and treatment services. This includes intersectional structural barriers related to transphobic and racist policies, and social factors like gender‐based stigma and racial discrimination that continue to be pertinent in addressing HIV inequities. As such, surveillance systems that are able to report data disaggregated at the intersections of gender identity, race, ethnicity and other factors can illuminate intertwined, oppressive, systematic drivers of HIV inequities in transgender populations. Investments and initiatives like EHE [61] in the United States can be further expanded and are highly needed to prioritize and value transgender populations across funding structures, goals and outcome measures.

## COMPETING INTERESTS

The authors have no competing interests.

## AUTHORS’ CONTRIBUTIONS

DMT, BM, AF, DD and AD conceived of and conducted original studies and analyses. DMT and AR drafted the original manuscript, all authors reviewed, edited and approved the final manuscript.

## FUNDING

This work was supported by the National Institute of Allergy and Infectious Diseases of the National Institutes of Health through grant number F31AI152542 awarded to DMT. This work was also supported by the American Sexually Transmitted Diseases Association (ASTDA) and the Northwest Center for Public Health Practice at the University of Washington's School of Public Health. DD was supported by the National Institutes of Allergy and Infectious Disease (NIAID) grant UM1AI068617.

## Data Availability

Data sharing is not applicable to this article as no datasets were generated or analysed during the current study.
